# Preparation of MOF-Based Core-Shell Gel Particles with Catalytic Activity and Their Plugging Performance

**DOI:** 10.3390/gels9010044

**Published:** 2023-01-04

**Authors:** Fengbao Liu, Jinsheng Sun, Xiao Luo

**Affiliations:** 1 School of Petroleum Engineering, China University of Petroleum (East China), Qingdao 266580, China; 2 School of Chemical and Environmental Engineering, Yangtze University, Wuhan 430100, China

**Keywords:** plugging, core-shell gel particles, metal-organic framework materials, molecular sieve catalytic activity, nanoscale crystals

## Abstract

Drilling fluid systems for deep and ultra-deep wells are hampered by both high-temperature downhole environments and lengthy cycle periods. Suppose that the gel particle-plugging agent, the primary treatment agent in the system, fails to offer durable and stable plugging performance. In such a scenario, the borehole wall is susceptible to instability and landslide after prolonged immersion, leading to downhole accidents. In this study, novel core-shell gel particles (modified ZIF) with ZIF particles employed as the core material and organosilicon-modified polyethylene polyamine (PEPA) as the polymer shell were fabricated using PEPA, in-house synthesized (3-aminopropyl) triethoxysilane (APTS), and the ZIF-8 metal-organic framework (MOF) as the raw materials to enhance the long-term plugging performance of gel plugging agents. The modified ZIF particles are nanoscale polygonal crystals and differ from conventional core-shell gel particles in that they feature high molecular sieve catalytic activity due to the presence of numerous interior micropores and mesopores. As a result, modified ZIF exhibits the performance characteristics of both rigid and flexible plugging agents and has an excellent catalytic cross-linking effect on the sulfonated phenolic resin (SMP-3) and sulfonated lignite resin (SPNH) in drilling fluids. Consequently, a cross-linking reaction occurs when SMP-3 and SPNH flow through the spacings in the plugging layer formed by the modified ZIF particles. This increases the viscosity of the liquid phase and simultaneously generates an insoluble gel, forming a particle-gel composite plugging structure with the modified ZIF and significantly enhancing the long-term plugging performance of the drilling fluid.

## 1. Introduction

As the exploration and development of oil and gas resources in the Tarim Oilfield gradually advance to deeper formations, the proportion of deep and ultra-deep wells grow annually [1,2,3]. The downhole high-temperature conditions and long cycle time requirements provide significant hurdles for the drilling fluid system’s treatment agent. Moreover, deep formations contain a high proportion of hard, brittle rocks. If the plugging agent fails to effectively block the seepage channels in the formation, it is likely to cause wellbore instability and landslide, as well as drilling sticking and burial, which impedes the progress of drilling operations [4,5,6,7].

To accomplish long-term, effective plugging of seepage channels, notably micro-pores and -channels in the formation, rigid and flexible plugging agents are generally employed simultaneously. In recent years, ultrafine calcium carbonate, nanoscale silicon dioxide, titanium dioxide, and zinc oxide have been utilized as rigid agents. The flexible agent mostly comprises modified asphalt and synthetic polymer gel particles [8,9,10]. Upon entering the seepage channels with the drilling fluid, the rigid particles are settled by the throat-blocking effect, initially filling the pores/channels and providing skeleton support. In contrast, the flexible plugging agent with good deformability and film-forming characteristics fills the pores between the rigid particles and particles and the channel wall, improving the density of the plugging layer and further blocking the seepage channels [11,12,13,14]. The capacity of rigid plugging agents to migrate into irregular microfractures is, however, severely constrained by the intrinsic agglomeration propensity of microscopic particles and their rigidity, which does not permit deformation. In this scenario, the skeleton support effect is inadequate, making it challenging for the flexible plugging agent to produce a thick plugging layer, resulting in poor long-term plugging performance.

Recently, a novel form of plugging material known as core-shell particles has emerged. The internal core consists of a rigid particle, such as nanoscale silica and ultrafine calcium carbonate, whose surface is modified by grafting functional monomers to generate an exterior flexible polymer coating layer. Thus, the functions of both rigid and flexible plugging agents are integrated [15,16]. Based on their synergistic action, the external flexible coating layer prevents the core-shell particles from surface energy effect-induced agglomeration/settling and maintains their agglomeration stability via the steric hindrance effect, a hydrophobic effect ascribed to the hydrophobic groups in the polymer, and electrostatic repulsion resulting from the dissociation of siloxane groups into silicon hydroxyl groups, when circulated with the drilling fluid. On the other hand, upon flowing into the seepage channels with the drilling fluid, the core-shell particles settle at the pore throat facilitated by the internal non-deformable rigid particles with the pH of the particle environment dropping to nearly neutral levels due to the excessive contact of the drilling fluid with the formation fluid. This weakens the repulsive effect between hydrophobic groups and silicon hydroxyl groups significantly, which no longer prevents neighboring particles from aggregating. Under the influence of hydrostatic column pressure differential and capillary force, the exterior flexible coating layer deforms and expands, generating a dense and robust plugging layer [17,18]. In addition, the silicone hydroxyl groups on the particle surface condense with the silicone hydroxyl groups in the clay component of the channel walls to form silicether bonds, eventually forming a dense and firm silicone gel plugging layer [19,20,21] to guarantee the particles’ long-term plugging performance.

Despite the aforementioned benefits of core-shell gel particles, as the well depth increases, the polymer coating layer becomes prone to thermal degradation due to the long-term high-temperature conditions of the downhole, resulting in a reduction in plugging performance [22,23,24]. To maintain sustainable drilling operations, it is vital to enhance the long-term plugging performance of these materials. Water-based drilling fluids for deep-well drilling are predominantly polysulfide systems; the two main treating agents, sulfonated phenolic resin (SMP-3) and sulfonated lignite resin (SPNH), have excellent temperature resistance and undergo subsequent cross-linking reactions to form block gels in the high-temperature downhole environment, which is conducive for resisting thermal degradation and preserving their polymeric properties [25,26,27,28,29]. Based on their properties, if both SMP-3 and SPNH can be incorporated into the formation of the plugging layer, it is anticipated that the sustainable plugging performance of the plugging agent would be improved without the addition of other agents or an increase in agent concentration.

The downhole cross-linking mechanism of SMP-3 and SPNH is such that the active hydrogen on the phenolic ring exhibits significant electrophilicity in a strongly alkaline environment of the drilling fluid and easily undergoes dehydration condensation with the hydroxymethyl group on the adjacent phenolic ring to form a phenolic ring connected by a stable methylene bridge, resulting in a high molecular weight of the polymer, an increase in viscosity of the system, and gelation or solidification under a high degree of cross-linking. The cross-linking reaction can be accelerated by raising the pH and mineralization level, as well as the temperature and reaction time [30,31,32]. However, in the seepage channels, the drilling fluid is significantly diluted by the neutral formation fluid, resulting in a significant reduction in pH, drastically lowering the electrophilic effect-based reaction activity between the active hydrogen and the hydroxymethyl group in SMP-3 and SPNH, thereby inhibiting the liquid phase viscosity increment, filtration loss reduction by cross-linking, and the generation of an insoluble gel to participate in the formation of the plugging layer [33,34,35,36]. Therefore, for SMP-3 and SPNH to participate in the formation of the plugging layer, it is important to increase their cross-linking reaction activity in a neutral environment.

Metal-organic framework (MOF) materials are a class of crystalline porous framework materials with a multidimensional periodic network structure generated by the self-assembly of metal ions or metal cluster units with organic ligands via coordination interactions [37,38]. They appear as microscopic or nanometric crystal particles with well-developed pore structures and large specific surface areas [39,40]. Based on these characteristics, MOFs have highly effective molecular sieve catalytic properties, with some containing transition metals with Lewis acid properties, equipping them with Lewis acid catalytic activity. Furthermore, the framework configuration of MOFs allows for the easy incorporation of hydrophilic polymeric macromolecules into their structures and increasing structural defects appropriately can significantly improve their suspension stability in the aqueous phase while simultaneously enhancing their catalytic activity [41,42,43,44]. Zeolitic imidazolate frameworks (ZIFs) are a significant subclass of MOFs that predominantly employ transition metals, such as Co, Zn, Fe, and Ni, as the central ion and imidazole or its derivatives as bidentate bridging ligands. The angle of the coordinate covalent bond formed is similar to that of Si–O–Si in the zeolite. Therefore, the topology and pore structure of ZIFs are comparable to those of zeolites, and ZIFs possess not only excellent structural strength but also exceptional temperature resistance [45,46,47]. Experiments demonstrated their superior structural stability in both strong alkaline and neutral conditions, where the ZIF framework improved the thermal and chemical stability of the introduced polymer [48,49,50,51]. For example, Dai et al. discussed the recent advances in ZIF-8-derived composites with outstanding stability for adsorption and photocatalytic wastewater pollutant removal [52]. Chen et al. synthesized ZIF-8 membranes on polyvinylidene fluoride (PVDF) hollow fiber, and excellent separation performances were obtained [53].

If the chemically inert rigid particles in the core-shell particles can be replaced with suitable ZIFs and suitable polymers can be incorporated into their framework structures as flexible coating layers, then novel core-shell gel particles with ZIFs as the cores can be synthesized. Its performance when circulated with the drilling fluid is identical to that of standard materials. However, when it flows into the seepage channels and forms a plugging layer, the ZIF core induces the cross-linking reaction between SMP-3 and SPNH in the drilling fluid as it passes through the interparticle spacings. This raises the viscosity of the liquid phase, and the strongly cross-linked insoluble material simultaneously forms a particle-gel composite plugging layer with the core-shell particles, resulting in a dense and durable plugging performance.

In this study, in-house synthesized (3-aminopropyl) triethoxysilane (APTS) and polyethylene polyamine (PEPA) underwent an addition reaction to produce an organosilicon-modified PEPA polymeric macromolecule, which was then introduced into the framework structure of the in-house prepared ZIF-8 via a post-synthetic modification to obtain the final polymer-modified ZIF product. Modified ZIF has superior thermal and chemical stability, is not prone to agglomeration, and can catalyze the cross-linking reaction between SMP-3 and SPNH to form a gel even in a neutral environment, thereby increasing the viscosity of the liquid phase and promoting the formation of a composite plugging layer by highly cross-linked insoluble matter and modified ZIF to ensure a durable plugging performance. By substituting the original plugging agents with modified ZIFs, the rheological properties of drilling fluids are successfully tuned, and its filtration loss reduction performance can be significantly enhanced.

## 2. Results and Discussion

### 2.1. Physicochemical Characterization of Modified ZIF

#### 2.1.1. SEM Analysis of the Modified ZIF

**Figure 1:**
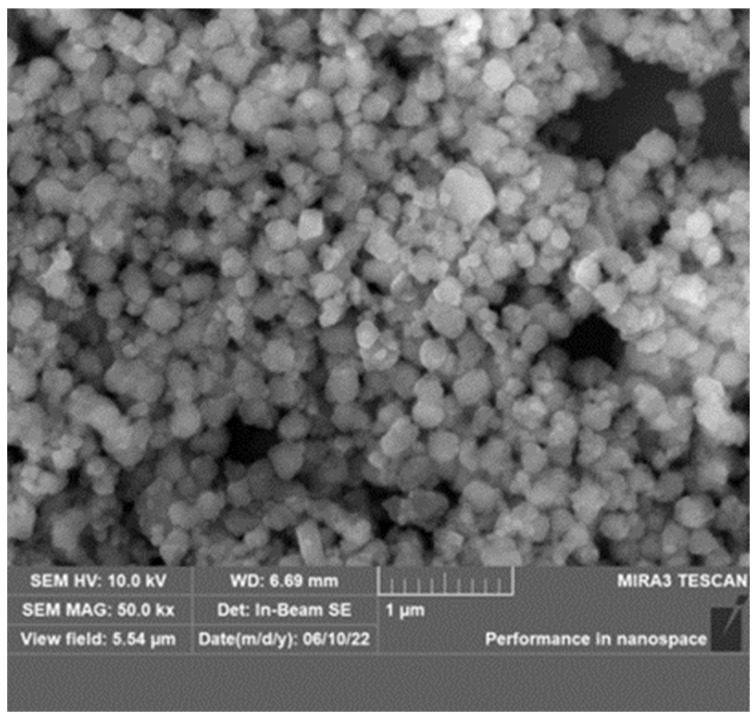
SEM image of the modified ZIF.

According to Figure 1, the morphology of the prepared modified ZIF is a highly regular prismatic polyhedron with a particle size in the region of 150–200 nm. Both the crystal form and particle size exhibit strong uniformity. The SEM image reveals that the modified ZIF particles are nanoscale particles, facilitating their penetration into the formation’s microfine seepage channels.

#### 2.1.2. XRD Analysis of the Modified ZIF

**Figure 2:**
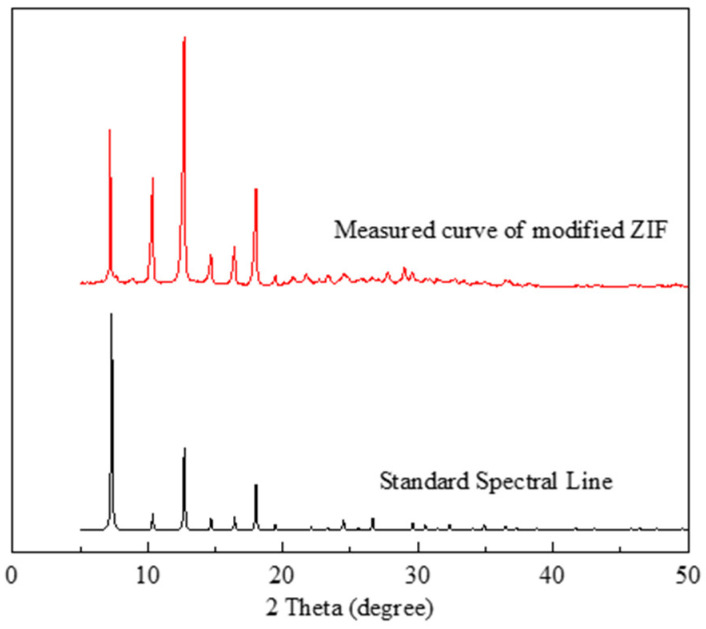
XRD pattern of the modified ZIF.

**Figure 3:**
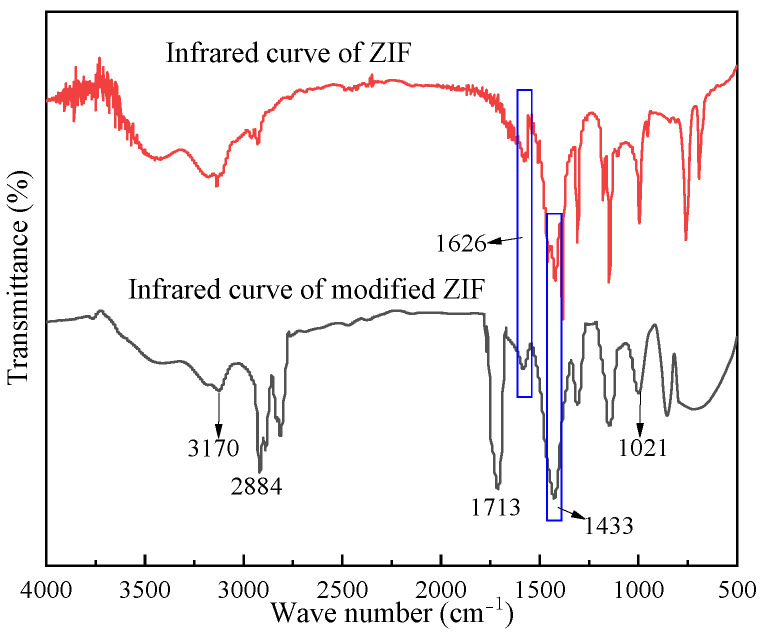
FTIR spectra of ZIF and modified ZIF.

The observed XRD pattern (Figure 2) of the modified ZIF reveals strong, substantial, and sharp distinctive peaks that are in excellent agreement with the typical diffraction peaks in the standard spectral line of ZIF-8 [54]. This suggests that the modified ZIF and ZIF-8 are consistent. Combining the preceding results with the morphology observed by SEM (Figure 3), it is evident that the synthesized ZIF does not lose its crystallinity as a result of the introduction of polymer macromolecules and, thus, retains good crystallinity.

#### 2.1.3. FTIR Analysis of the Modified ZIF

As shown in Figure 3, the same characteristic peaks at 1626 and 1433 cm^−1^ appear in both FITR spectra of the ZIF and modified ZIF, attributed to the bending vibration of N–H and C–N in the 2-methylimidazole ring, respectively. The main difference between the two FITR spectra is that the FITR spectrum of the modified ZIF exhibit characteristic peaks of N–H in the primary amine group, C=O in the amide group, and Si–O in the siloxane group at 3170, 1713, and 1021 cm^−1^, respectively, and the characteristic peak of -CH3 at 2884 cm^−1^ intensified and became significant. This confirms the successful introduction of organosilicon-modified PEPA macromolecules into the ZIF framework in the modified ZIF, and the molecular structure of the modified ZIF is consistent with our expectations.

### 2.2. Performance Study of the Modified ZIF in Drilling Fluid

#### 2.2.1. Simulated Liquid Phase Environment

Since the modified ZIF is exposed to prolonged high-temperature conditions in the strongly alkaline and highly mineralized drilling fluid before entering the seepage channels of the formation, it must have excellent structural stability to maintain its catalytic activity and thus promote the cross-linking reaction between SMP-3 and SPNH for gel formation. To formulate a typical polysulfide drilling fluid system, a deionized aqueous solution containing 7 wt% KCl was synthesized, and its pH was adjusted to 11 using NaOH. The aqueous solution was utilized to simulate the liquid phase environment for the modified ZIF, and a certain amount of the modified ZIF was added to the solution to form a suspension. The mixture was subsequently aged at 200 °C for 32 h to investigate the physicochemical properties of the modified ZIF after aging.

#### 2.2.2. SEM Analysis of the Modified ZIF after Aging

**Figure 4:**
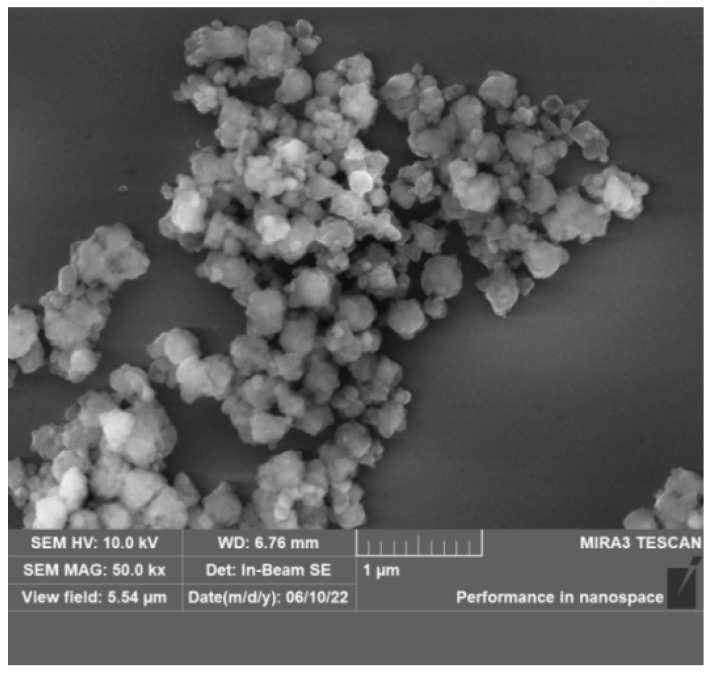
SEM images of the modified ZIF after aging.

The modified ZIF particles are predominantly 150–300 nm in size and retain their typical polygonal crystal morphology (Figure 4), demonstrating that the modified ZIF can still retain its nanoparticle properties and structure after aging.

#### 2.2.3. XRD Analysis of the Modified ZIF after Aging

**Figure 5:**
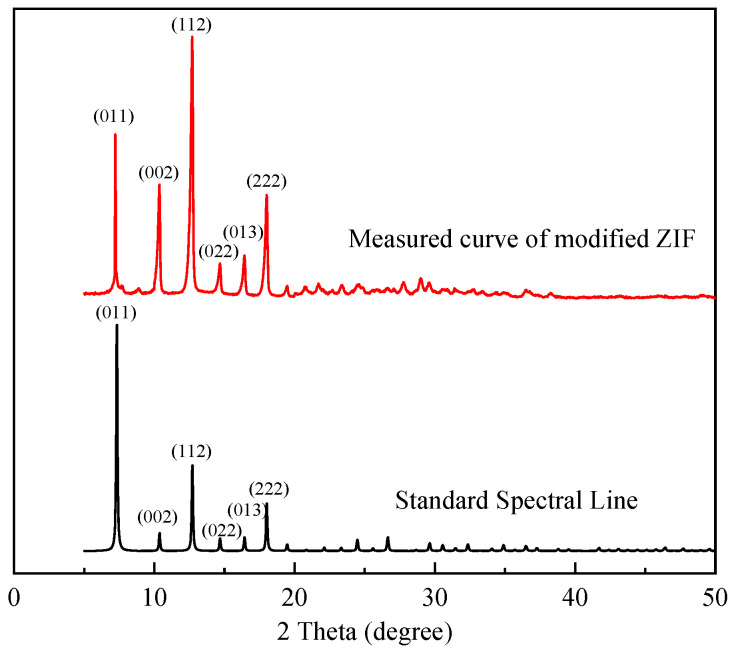
XRD patterns of modified ZIF before and after aging at 200 °C for 32 h.

The XRD patterns of modified ZIF before and after aging at 200 °C for 32 h are compared in Figure 5. Significant crystalline characteristic peaks are still present in the XRD pattern of the modified ZIF after 32 h of aging at 200 °C, and the peak positions are essentially the same as before aging, indicating that the aged ZIF particles are still crystals with high crystallinity and their structures have not collapsed.

#### 2.2.4. Particle Size Distribution of Modified ZIF after Aging

**Figure 6:**
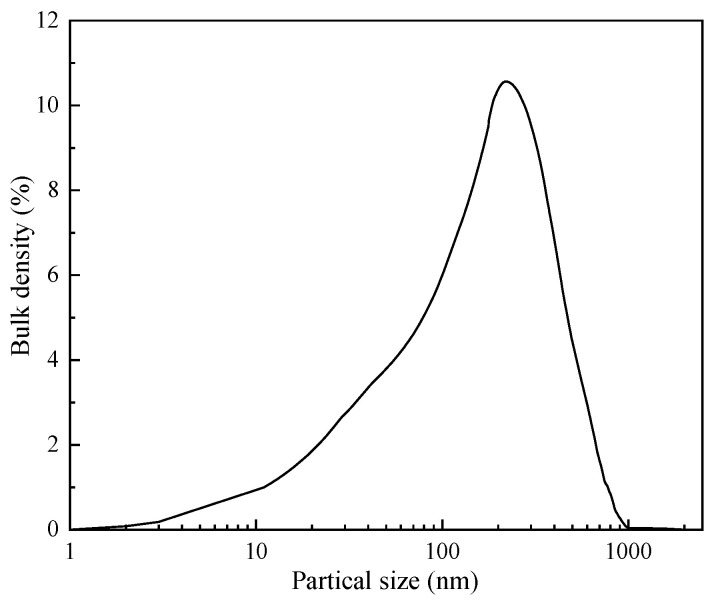
Particle size distribution of modified ZIF after aging.

As depicted in Figure 6, the particle size of the aged modified ZIF is highly concentrated in the range of 150–300 nm, indicating that the particles do not agglomerate under high temperatures and remain as typical nanoscale particles, which is conducive to their migration into microfine seepage channels to achieve the “sealing tail” plugging effect.

#### 2.2.5. BET Analysis of Modified ZIF

**Figure 7:**
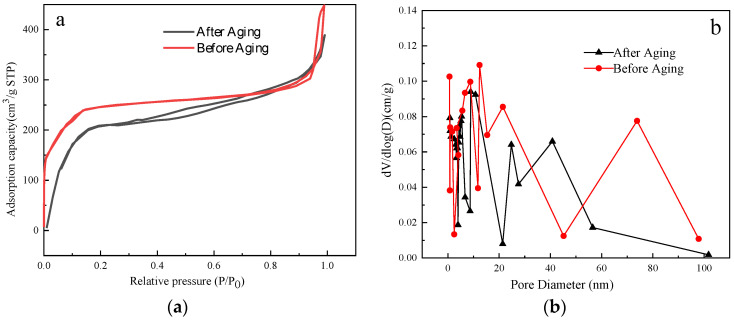
(**a**) Adsorption–desorption isotherm and (**b**) pore size distribution of ZIF before and after aging.

If the ZIF derivative plugging agent is to retain its catalytic activity in the high-temperature downhole environment, it must retain its molecular sieve properties after prolonged exposure to high-temperature and high-salinity conditions. Therefore, the modified ZIF was subjected to BET analysis, and the adsorption–desorption isotherm and pore structure were examined. Figure 7 demonstrates the results. The Brunauer–Emmett–Teller (BET) surface area of modified ZIF before and after is 1125.6291 m^2^/g and 998.6053 m^2^/g, respectively, indicating the specific surface area of the material was not significantly reduced after aging. Furthermore, the pore size distribution (PSD) confirms that the modified ZIF after aging still has a porosity that consists of plenty of micropores and mesopores.

#### 2.2.6. Catalytic Performance of the Modified ZIF on SMP-3 and SPNH

To study the catalytic performance of the modified ZIF on the cross-linking reaction between SMP-3 and SPNH in a pH-neutral environment after contacting the formation fluid, a deionized water solution of 7.0 wt% KCl was prepared, followed by the addition of 3 wt% SMP-3 and 3 wt% SPNH, and then 4 wt%-modified ZIF was added to form a suspension after stirring. The suspension was aged for 32 h at 200 °C. After that, the suspension was vacuum-filtered to separate the insoluble material from the filtrate. The insoluble material was then rinsed with excess deionized water and resuspended by stirring in deionized water. The suspension was then filtered and separated a second time. The process was performed three times, and the resulting substance was pure and insoluble. The relevant physicochemical parameters of the insoluble matter and the filtrate from the initial separation were investigated.

#### 2.2.7. FITR Spectra of Insoluble Matter

**Figure 8:**
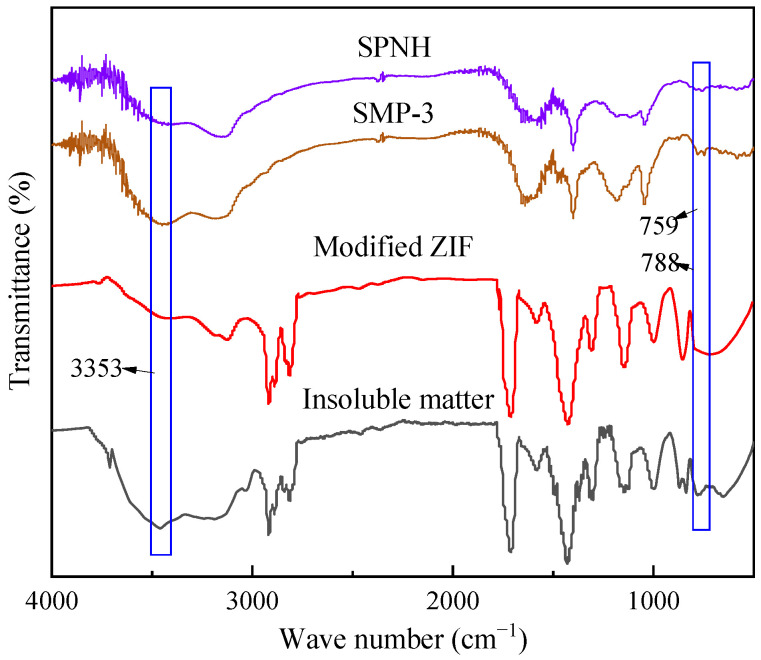
FITR spectra of SPNH, SMP-3, modified ZIF, and insoluble matter.

As shown in Figure 8, after 32 h of aging in the simulated liquid phase, the FITR spectra of the insoluble matter are highly consistent with that of the modified ZIF, demonstrating that modified ZIF is the primary component of the insoluble matter. However, the former exhibits three peaks at 3353, 759, and 788 cm^−1^ that are absent in the latter; these three peaks are consistent with the stretching vibration peaks of associate hydrogen bonds and the bending vibration peaks of disubstituted and trisubstituted benzene in the FITR spectra of SPNH and SMP-3, respectively. This verifies the excessive cross-linking reaction between SPNH and SMP-3 catalyzed by the modified ZIF to produce gels, as well as an integrated particle-gel composite structure in conjunction with the modified ZIF.

#### 2.2.8. Gel Permeation Chromatography (GPC) Determination of the Filtrate

**Figure 9:**
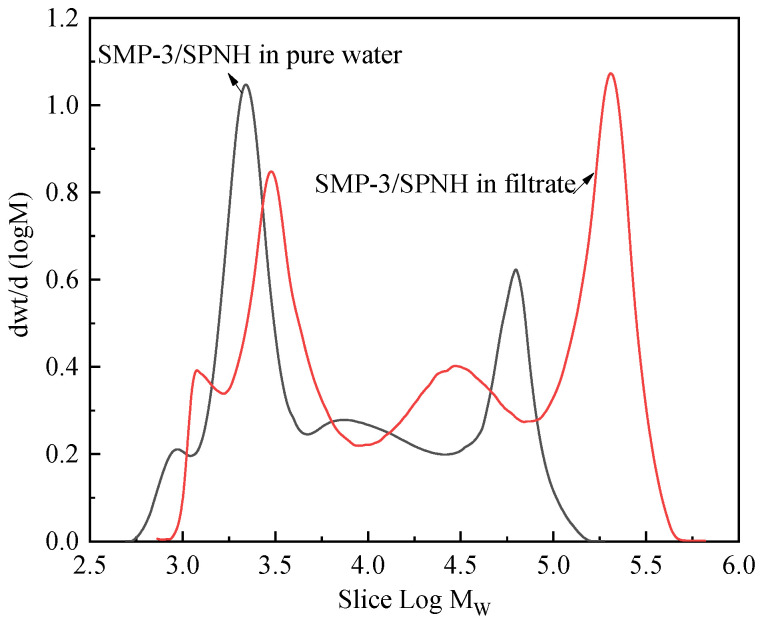
GPC spectra of SMP-3/SPNH aged in the 7.0 wt.% KCl deionized aqueous solution and the filtrate.

The molecular weights of the identical amount of SMP-3/SPNH mixtures aged in a 7.0 wt.% KCl deionized aqueous solution and in the filtrate after the initial separation were evaluated using GPC. The results are depicted in Figure 9 and Table 1.

As shown in Figure 9, the GPC spectrum of the SMP-3/SPNH mixture aged in the filtrate exhibited a significant overall rightward shift relative to that of the SMP-3/SPNH mixture aged in the KCl solution. The peak appears on the right side of a larger molecular weight, indicating that the molecular weight of the SMP-3/SPNH mixture aged in the filtrate is greater than that aged in the KCl solution. As shown in Table 1, the weighted average molecular weight (Mw), number average molecular weight (Mn), and peak molecular weight (Mp) of the mixture after aging in the KCl solution are 3.89 × 104, 3.65 × 105, and 2.93 × 104, respectively. In contrast, the Mw, Mn, and Mp of the mixture after aging in the filtrate are 6.65 × 104, 7.12 × 105, and 18.74 × 105, representing a significant increase. It demonstrates that the modified ZIF can catalyze the cross-linking reaction between SMP-3 and SPNH in a neutral environment, resulting in a considerable rise in the gels’ molecular weight.

Combining the results of Section 2.2.7 and Section 2.2.8, the modified ZIF can induce the cross-linking of SMP-3 and SPNH in a neutral environment, resulting in a rise in their molecular weights and the degree of cross-linking. The water-soluble matter with a low degree of cross-linking can increase the viscosity of the filtrate, thereby increasing its flow resistance, whereas SMP-3 and SPNH with a high degree of cross-linking form insoluble gels, forming a particle-gel composite structure with the modified ZIF, which is conducive to improving the plugging performance.

#### 2.2.9. Performance Evaluation of the Modified ZIF-Containing Drilling Fluid

Two formulations of field-typical polysulfide drilling fluids were chosen for evaluation: (1) 2% bentonite + 0.3% NaOH + 3% SMP-3 + 3% SPNH + 6% viscosity-reducing agent SMC + 4% ultrafine calcium carbonate + 2% modified oxidized asphalt TYJS-1 + 1% bituminous lignite HLQ-2 + 7% KCl + 1% oil-based lubricant EPL-1, and (2) 3.5% bentonite + 0.5% NaOH + 3% SMP-3 + 3% SPNH + 4% ultrafine calcium carbonate + 4% sulfonated asphalt FY-1A + 7% KCl + 2% extreme pressure lubricant HY-202. Using barite, both systems were weighted to 2.0 g/cm^3^ density. In system 1, the plugging agents are ultrafine calcium carbonate, TYJS-1, and HLQ-2. In system 2, the plugging agents are ultrafine calcium carbonate and FY-1A. The blocking agents in the two systems were replaced with 5 wt% modified ZIF, and the performance of the drilling fluid systems before and after the replacement was evaluated prior to and after aging at 200 °C for 16, 32, and 48 h, respectively. Table 2 presents the results.

As demonstrated in Table 2, the rheology and filtration loss reduction of both drilling fluid systems prior to aging remained identical after replacing the original plugging agents with 4% modified ZIF. After aging at 200 °C, the apparent viscosity, plastic viscosity, and dynamic shear force of the drilling fluids prior to replacement decrease significantly as the aging duration increases, while API and HTHP filtration losses increase gradually. The API filtration losses of the two drilling fluids before replacement increased by 38.4% and 58.3% after 48 h of aging, whereas the HTHP filtration losses increased by 30.8% and 42.1%, respectively. In addition, the thickness of the API and HTHP mud cake also increased significantly. The rheological properties of the drilling fluids after replacement and aging are consistent with their properties before aging; not only did they not show a significant decrease, but the apparent viscosity, plastic viscosity, and dynamic shear force of system 1 after aging for 48 h were marginally greater than those prior to aging. This suggests that SMP-3 and SPNH in the system cross-linked under the catalytic action of the modified ZIF, generating a gel structure to enhance the molecular weight and thus maintain the viscosity of the system. Similarly, the API and HTHP filtration losses remained unchanged prior to and after aging for systems with modified ZIFs. The thickness of the API mud cake only increased from 0.5 to 0.6 mm and that of the HTHP cake from 1.2 and 1.4 mm to 1.4 and 1.6 mm, respectively, after 48 h of aging. The aforementioned results suggest that the modified ZIF has no negative effect on the rheological properties of drilling fluids and retains its catalytic activity despite being exposed to drilling fluids at elevated temperatures for extended periods of time. By cross-linking SMP-3 and SPMH in the system, it is possible to prevent the system from exhibiting a considerable drop in viscosity after prolonged exposure to high temperatures. In addition, the insoluble matter with a high degree of cross-linking forms a particle-gel composite plugging layer with the modified ZIF, considerably improving the drilling fluids’ long-term plugging performance.

## 3. Conclusions

Organosilicon-modified PEPA was synthesized via an addition reaction of PEPA with an in-house prepared APTS organosilicon monomer. It was then employed to conduct a post-synthetic modification reaction of an in-house synthesized ZIF-8, resulting in the formation of core-shell particles (modified ZIF) with the ZIF particles serving as the core material and the organosilicon-modified PEPA as the shell coating layer. The modified ZIF particles are nanoscale crystal particles with an average particle size between 150 and 200 nm and have excellent thermal and chemical stability in the drilling fluid. After 32 h of aging at 200 °C, the particles maintained their nanoscale polygonal crystal structure without agglomeration and had more interior micropores and mesopores in addition to high theoretical catalytic activity. While increasing the viscosity of the liquid phase, the modified ZIF can catalyze the cross-linking reaction between SMP-3 and SPNH in a neutral environment for gel formation. Furthermore, the insoluble matter with a high degree of cross-linking forms a particle-gel composite structure with the modified ZIF to improve its plugging performance. By replacing the original plugging agents with the modified ZIF, the rheological properties of the drilling fluid can be effectively tuned. Long-term high-temperature action does not result in a considerable decrease in the viscosity of the drilling fluid, thereby significantly improving the filtration loss reduction of the drilling fluid compared to the traditional plugging agent.

## 4. Materials and Methods

### 4.1. Preparation of Modified ZIF

**Figure 10:**
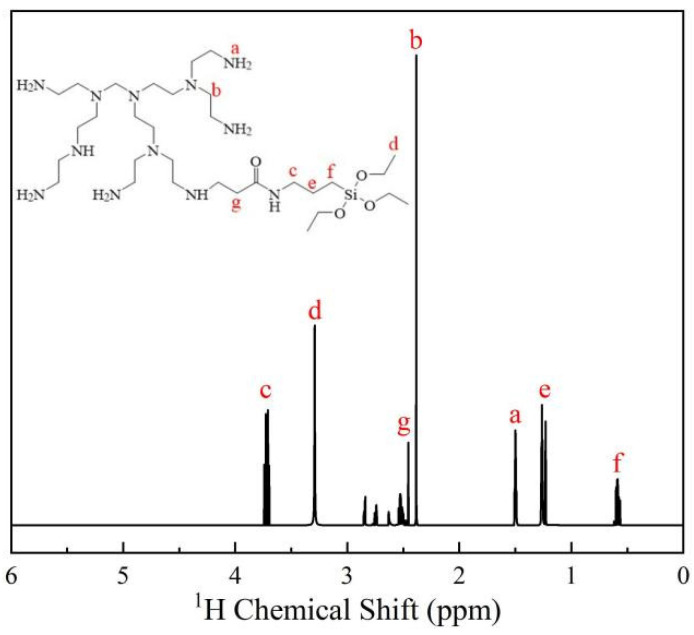
^1^H NMR spectrum of the organosilicon-modified PEPA.

First, 27.52 g of APTS (preparation method reference) was dissolved in excess anhydrous ethanol, mixed well, and poured into a three-necked flask. The solution was then heated slowly to 65 °C while being agitated under a protected N_2_ atmosphere. Then, dropwise additions of anhydrous ethanol containing 73.62 g PEPA dissolved in advance were made. The total concentration of raw materials in the mixture was 20 wt.%, and the reaction was kept for 24 h under N_2_ protection to produce a light-yellow viscous liquid. Next, rotary evaporation was used to remove the anhydrous alcohol from the reaction system, after which the residue was dissolved in excess acetone to remove unreacted APTS, followed by rotary evaporation to remove the acetone. The light-yellow viscous liquid obtained after three repetitions of the preceding procedure was organosilicon-modified PEPA. The product’s ^1^H NMR spectrum is depicted in Figure 10.

As depicted in Figure 10, the characteristic shift of the primary amine proton at 1.53 ppm, the chemical shift of the proton in the methylene group bonded to the carbonyl group at 2.37 ppm, the characteristic shift of the methyl proton in the Si–OCH3 group at 3.35 ppm, and the chemical shift of the methylene proton bonded to the Si in the Si–OCH3 group at 0.56 ppm indicate that the molecular structure of the prepared product is as expected [50].

**Figure 11:**
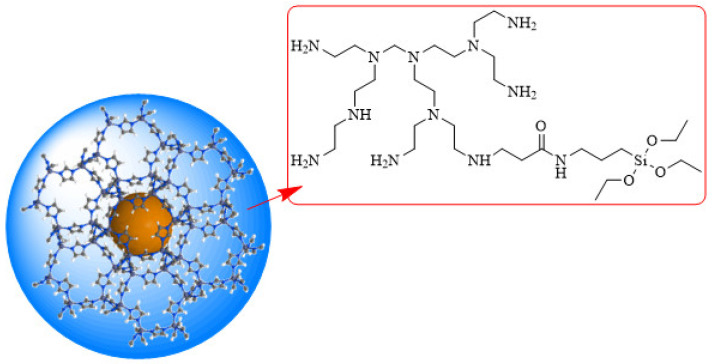
Schematic structure of the modified ZIF.

For the subsequent preparation of modified ZIF, 12.73 g of ZIF-8 (preparation method reference) was dissolved in 75 mL of anhydrous ethanol, and 27.29 g of organosilicon-modified PEPA pre-dissolved in excess anhydrous ethanol was added to the mixture while stirring. The temperature was increased to 60 °C while stirring continuously, and the reaction was then held for 24 h. The reaction system was then subjected to rotary evaporation under reduced pressure to remove a portion of the anhydrous ethanol, followed by centrifugation to extract the crude product. To collect the insoluble material, the crude product was next washed with deionized water and anhydrous ethanol in succession. The washing process was performed three times, and then the product was vacuum-dried. The resultant white powder was polymer-modified ZIF (henceforth referred to as modified ZIF), whose structure is depicted schematically in Figure 11.

### 4.2. Structural Characteristics

Fourier transform infrared spectra (FT-IR) were obtained with a Nicolet 6700 spectrophotometer. The total specific surface area and nanopore size distribution of the obtained products were measured by a Brunauer–Emmett–Teller (BET) nitrogen adsorption-desorption test at −196 °C under nitrogen. The crystal type of the synthesized ZIF samples was characterized by X-ray diffraction (XRD) in the 2θ range of 10~50°.

## Figures and Tables

**Table 1:** Molecular weights of SMP-3/SPNH aged in the 7.0 wt.% KCl deionized aqueous solution and the filtrate.

Sample	State	M_n_	M_w_	M_p_	Polydispersity
SMP-3/SPNH Mixture	Solution	3897	36,544	2927	9.378
	Filtrate	6651	71,235	187,374	10.712

**Table 2:** Performance tests of drilling fluids before and after replacement.

Drilling Fluid	Scenario	*AV*/(mPa·s)	*PV/*(mPa·s)	*YP/*Pa	*FL*(API)/mL	*FL*(HTHP)/mL
(1) Before replacement	Before aging	56.5	43.0	13.5	2.6/0.5 mm	-
	Aging for 16 h	44.5	34.0	10.5	3.2/0.5 mm	7.8/1.6 mm
	Aging for 32 h	41.5	34.0	7.5	3.4/0.8 mm	8.6/1.6 mm
	Aging for 48 h	37.5	31.0	6.5	3.6/0.8 mm	10.2/2.2 mm
(1) After replacement	Before aging	53.5	42.0	11.5	2.4/0.5 mm	-
	Aging for 16 h	51.5	40.5	11.0	2.4/0.6 mm	7.0/1.2 mm
	Aging for 32 h	53.5	41.0	12.5	2.2/0.6 mm	7.4/1.4 mm
	Aging for 48 h	56.5	43.0	13.5	2.4/0.6 mm	7.4/1.4 mm
(2) Before replacement	Before aging	57.0	46.0	11.0	2.4/0.5 mm	-
	Aging for 16 h	44.5	35.5	9.0	3.2/0.7 mm	7.6/1.4 mm
	Aging for 32 h	42.5	35.0	7.5	3.4/0.8 mm	8.8/1.8 mm
	Aging for 48 h	39.5	33.0	6.5	3.8/0.8 mm	10.8/2.2 mm
(2) After replacement	Before aging	56.5	43.0	13.5	2.4/0.5 mm	-
	Aging for 16 h	49.5	35.5	14.0	2.6/0.5 mm	7.0/1.4 mm
	Aging for 32 h	52.5	41.0	11.5	2.2/0.5 mm	7.2/1.4 mm
	Aging for 48 h	54.5	43.0	11.5	2.2/0.6 mm	6.8/1.6 mm

## Data Availability

All persons included have agreed to confirm.
